# Benzalkonium Chloride-Induced Nephrotoxicity in 2D Cultures and a Human Kidney-on-a-Chip System

**DOI:** 10.1021/acs.est.5c15625

**Published:** 2026-05-25

**Authors:** Marie N. Brzoska, Ryan P. Seguin, James W. MacDonald, Jade Yang, Vanessa A. Lopez, Sydney Arnzen, Edward J. Kelly, Libin Xu

**Affiliations:** † Department of Medicinal Chemistry, 7284 University of Washington, Seattle, Washington 98195, United States; ‡ Department of Pharmaceutics, 7284 University of Washington, Seattle, Washington 98195, United States; § Department of Environmental and Occupational Health Sciences, 7284 University of Washington, Seattle, Washington 98195, United States

**Keywords:** benzalkonium chloride, proximal tubule epithelial cell, microphysiological system, kidney-on-a-chip, nephrotoxicity

## Abstract

Benzalkonium chlorides (BACs) are antimicrobial compounds widely used across a variety of settings, and the COVID-19 pandemic has led to significantly increased usage of BAC-containing products. Previous studies in animals have shown that the kidney is the primary site of BAC accumulation, whereas the liver does not accumulate BACs, likely due to efficient hepatic cytochrome P450-mediated metabolism. Thus, we hypothesized that low BAC-metabolizing capacity contributes to the extent of BAC buildup in the kidney and promotes subsequent kidney injury. Using 2D-cultured proximal tubule epithelial cells (PTECs), we found that renal metabolism is insufficient to detoxify BACs. We then assessed BACs’ nephrotoxicity using a novel 3D “kidney-on-a-chip” microphysiological system (MPS). The MPS was exposed to C_12_- or C_14_-BAC at 100 nM for 1 week. Transcriptomic analysis of the 3D-cultured PTECs reveals several significantly altered biochemical pathways following BAC exposure, including focal adhesion and cholesterol biosynthesis. Additional studies utilizing orthogonal techniques validated the phenotypic changes associated with some of the significantly altered pathways using 2D-cultured PTECs. Together, these findings demonstrate that the poor BAC-metabolizing capacity of PTECs contributes to the nephrotoxicity of BACs.

## Graphical abstract

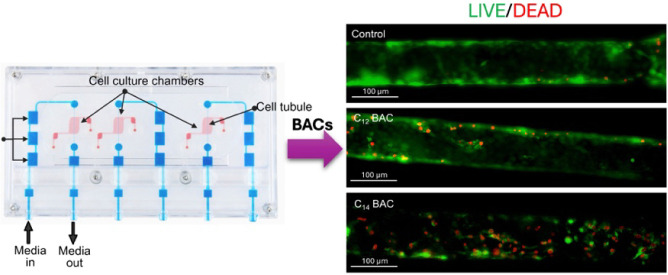

## Introduction

Benzalkonium chlorides (BACs) are quaternary ammonium compounds (QACs) widely used across various industries due to their broad-spectrum antimicrobial properties.^1,2^ BACs are amphiphilic molecules, consisting of a positively charged ammonium headgroup and a long aliphatic tail (Figure 1). Their mechanism of antimicrobial action involves perturbing and disrupting the lipid membrane.^1^ Due to BACs’ antimicrobial activity, they are active ingredients in disinfectant and cleaning solutions (sprays, wipes, and hand sanitizers), medical products (eye drops and nasal sprays), cosmetic and hygiene products, and sanitization tools in the agricultural and food-processing industries.^1^ Furthermore, BACs have been detected in food products, notably milk and milk-derived products.^3^ Major routes of exposure to BACs include ingestion, inhalation, and dermal/ocular/nasal contact.^4^ The COVID-19 pandemic led to increased use of cleaning and disinfectant solutions, resulting in higher indoor exposure to BACs and a 174% increase in BAC levels in human blood compared to prepandemic levels.^5,6^ Human feces samples have shown the presence of 14 unique QACs, including BACs, where relatively high levels of C_12_ BAC (mean = 212 nM; max = 384 nM) and C_14_ BAC (mean = 317 nM; max = 615 nM) were detected.^7^

**Figure 1:**
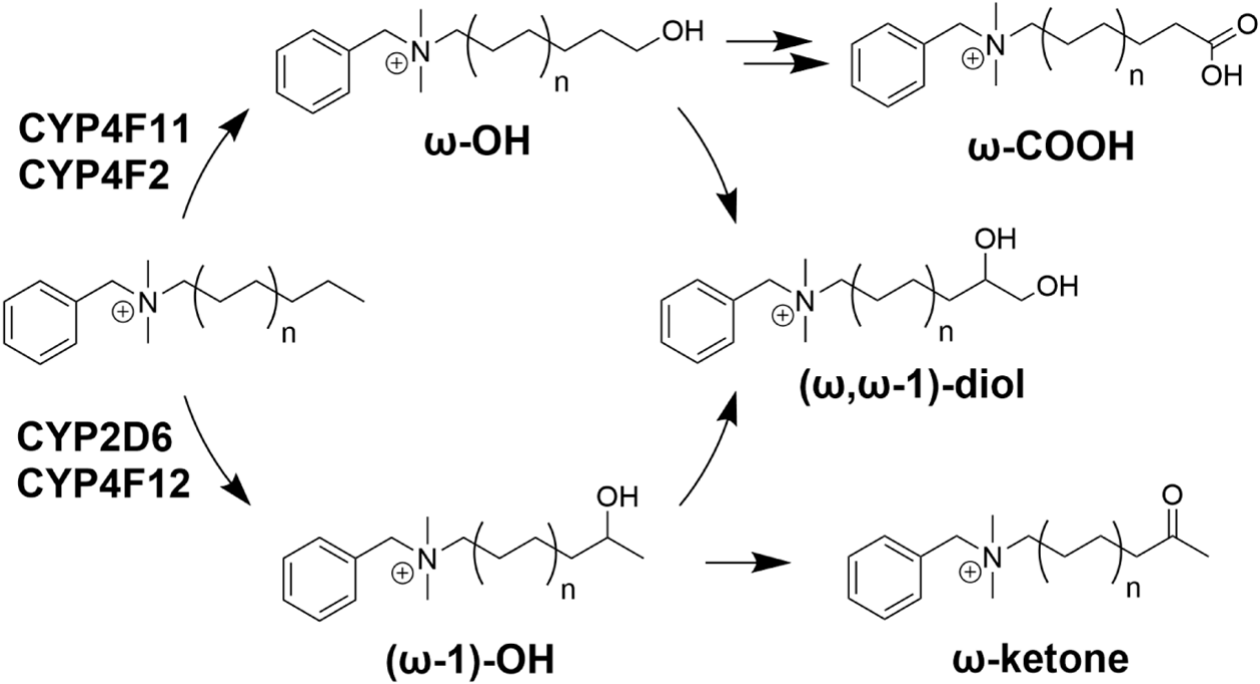
BAC metabolic pathways based on current knowledge.^25^

The FDA called for additional safety data on BAC use in consumer products due to its little-known effects on human health, especially under low-level, chronic exposure.^8^ Human clinical observations have demonstrated that toxicity can manifest physically through different routes of exposure, such as the development of occupational asthma in healthcare personnel using BAC-containing disinfectants^9,10^ or contact dermatitis from exposure to BAC-containing laundry products.^11^ After using ophthalmic solutions containing BACs as preservatives, glaucomatous patients experience allergic and dry-eye symptoms, including inflammation, redness, and burning.^12^ Additional adverse effects include decreased fertility and increased incidence of neural tube defects in mice,^13,14^ as well as altered mitochondrial function in human corneal epithelial primary cells, osteosarcoma cells, and human breast carcinoma cells.^15,16^ Our lab has also demonstrated that BACs significantly alter cholesterol and lipid homeostasis in vitro and in neonatal mouse brains, notably by inhibiting 3β-hydroxysterol-Δ^7^-reductase (DHCR7) in the cholesterol biosynthesis pathway.^17,18^

When administered orally or intravenously, BACs are widely distributed across major organs at a wide range of concentrations.^19,20^ In rats, a single intravenous dose of 7 mg/mL resulted in the highest accumulation of BACs in the kidney after 30 min (50 μg/g), while a single dose of 250 mg/kg by oral gavage resulted in the highest levels in most organs after 24 h: kidney (5.25 μg/g), lung (2.75 μg/g), liver (0.72 μg/g), and blood (0.34 μg/g).^19,20^ Circulating QACs can bind to serum proteins.^6,21–23^ However, due to their short plasma half-lives (1.8–3.7 h) and high volumes of distribution (5.6–6.7 L/kg),^19,20^ partitioning into tissues is favored over binding to serum protein.^2^ Liver accumulation is relatively low, likely due to the efficient metabolism of these compounds by cytochromes P450 (CYPs). Human CYP4F11 and CYP4F2 can mediate BAC metabolism to form ω-hydroxy metabolites, which can be further oxidized to carboxylic acid or diol metabolites. As an alternative route, CYP2D6 and CYP4F12 can produce ω-1-hydroxy products, which can subsequently form ketone or diol metabolites (Figure 1).^24,25^ It is known that the kidney expresses much lower levels of BAC-metabolizing CYP enzymes than the liver.^26–29^ We hypothesize that the low BAC-metabolizing capacity contributes to the extent of BAC buildup in the kidney and thus contributes to subsequent BAC-induced kidney injury.

New approach methodologies (NAMs) to replace animal models are becoming increasingly important in evaluating drugs and other xenobiotics. A human “kidney-on-a-chip” microphysiological system (MPS) is a NAM in which human primary proximal tubule epithelial cells (PTECs) are cultured in a 3D tubular structure that mimics the human proximal tubule.^30^ The 3D “kidney-on-a-chip” MPS is advantageous over 2D monolayer cultures because the 3D-cultured cells can polarize in response to shear forces generated by continuous flow. The MPS replicates the structures and functions of human kidney proximal tubules, including the polarized expression of protein markers of the luminal and basal membranes, as well as the functions of proteins critical to secretion and reabsorption processes, such as xenobiotic-metabolizing enzymes and transporters.^30^ The MPS also represents a superior model to animal models because humans and animals have different physiology and protein expression profiles. This kidney MPS has been successfully applied to the studies of nephrotoxicity induced by the antibiotic polymyxin B.^31^

This study aims to investigate the cellular metabolism, accumulation, and nephrotoxicity of BACs using complementary 2D-cultured PTECs and the human “kidney-on-a-chip” MPS. We first examined the impact of metabolism on the cytotoxicity of BACs in PTECs using a CYP4F inhibitor in 2D cultures. We then examine BAC toxicity in the kidney MPS using LIVE/DEAD cell staining and transcriptomic analysis of significantly altered pathways. Finally, we validated two of the significantly altered pathways, cholesterol biosynthesis and focal adhesion, using orthogonal assays.

## Materials and Methods

### Materials

Optima LC–MS grade acetonitrile (ACN), water, formic acid, and ammonium formate were purchased from Fisher Scientific (Santa Clara, CA). BACs of various alkyl chain lengths (C_8_-, C_10_-, C_12_-, C_14_-, and C_16_-BAC) and all synthetic reagents and starting materials were obtained from Sigma-Aldrich (St. Louis, MO). Deuterated BACs were synthesized as described in Herron et al.^17^ PTECs were isolated from human kidney tissue at the University of Washington Medical Center under a University of Washington Institutional Review Board-approved Human Subjects protocol and processed as described previously.^30^ All sterol standards were purchased from Avanti Polar Lipids.^32^

### Cell Isolation, Culture, and Seeding in the MPS Platform

PTECs from individual human donors (Table S1) were cultured, maintained, and harvested as previously described.^30,31^ The resulting cell pellet was suspended in cell media for 2D plating or injection into the 3D kidney-on-a-chip MPS. 2D cells were allowed to grow for several days to achieve ∼70% confluency before treatment.

### BAC Toxicity in 2D Culture

PTECs derived from Donor PT13 were cultured in 96-well plates (Corning, NY) treated with either vehicle control (0.1% dimethyl sulfoxide [DMSO]), C_12_-, C_14_-, or C_16_-BAC, and corresponding ω-OH metabolites (ω-OH-C_12_-BAC, ω-OH-C_14_-BAC, ω-OH-C_16_-BAC) (n = 3) at concentrations of 40, 20, 10, 5, 1, 0.5, 0.1, 0.05, and 0 μM for 48 h. Cells were incubated at 37 °C and 5% CO_2_ for 48 h, after which cell viability was assessed using the CellTiter 96 Aqueous One Solution Proliferation Assay (Promega; Madison, WI) according to the manufacturer’s protocol. Absorbance was recorded at 490 nm.

To evaluate the effect of HET0016 on BAC toxicity, donor PT11 was cultured in 96-well plates (Corning, NY) and treated with either vehicle control (0.1% DMSO) or C_12_-, C_14_-, and C_16_-BAC with or without 0.5 μM of HET0016 for 48 h (*n* = 3 for each group). BAC concentrations are as follows: 40, 10, 4, 1, 0.4, 0.1, 0.04, 0.01, and 0 μM. Cell viability was evaluated as described above.

### Extraction of BACs in Cells Cultured in 96-Well Plates

After aspirating the incubation media and briefly rinsing the cells with buffer, 100 μL of cell extraction solvent mixture (80% ACN/20% H_2_O containing the internal standard mix comprised of *d*_0_-C_6_-, *d*_0_-C_8_-, *d*_7_-C_10_, *d*_7_-C_12_, *d*_7_-C_14_, and *d*_7_-C_16_ BACs at 500 nM each) was added to the wells at room temperature. The resulting mixtures were incubated at room temperature for 15 min, then transferred to a clean 96-well sample-processing plate. To the original cell culture plate, an additional 100 μL of a 1:1 ACN:H_2_O solution was added. The additional 100 μL and remaining cell debris were then transferred to the sample processing plate, and these combined extracts were centrifuged at 4000 rpm at 4 °C for 15 min. Lastly, 30 μL of Optima H_2_O was added to a clean NUNC 96-well plate (Thermo Fisher Scientific, Grand Island, NY), and 120 μL of supernatant was transferred from the previous 96-well plate. The NUNC plates were sealed with a preslit silicon mat (Thermo Fisher Scientific, Grand Island, NY) and stored at 4 °C until LC–MS analysis.

### Kidney-on-a-Chip Preparation

Triple-channel MPS devices (Triplex; Nortis Inc.) were prepared according to previously described procedures.^30,33^ Briefly, the devices were filled with 6 mg/mL rat tail collagen type 1 (Ibidi, Madison, WI) at 4 °C and left at room temperature overnight to allow the extracellular matrix to solidify. Mandrels of the platforms were removed, and PTEC media was perfused through the hollow channels overnight at 1 μL/min. Channels were coated with mouse collagen IV at 0.1 mg/mL (Corning, NY) to facilitate PTEC adhesion, followed by injection of 2.5 μL of a PTEC suspension at 20 × 10^6^ cells/mL. Cells were left to adhere to the channels for 3–4 h in a 5% CO_2_ incubator, and the devices were subsequently perfused at 1 μL/min for several days before treatment. Kidney MPS that exhibited functional channels and appropriate cell density were used for treatment. All chip experiments used donor PT16.

### Kidney-on-a-Chip Treatment and Effluent Preparation for LC–MS

MPS were treated with either vehicle (0.1% DMSO), C_12_-BAC (100 nM), or C_14_-BAC (100 nM) for 1 week (*n* = 3 for each group). Chip effluents were collected every 24 h. Chip effluents were quenched using ACN:H_2_O (1:1) containing internal standards of *d*_0_-C_6_-, *d*_0_-C_8_-, *d*_7_-C_10_, *d*_7_-C_12_, *d*_7_-C_14_, and *d*_7_-C_16_ BACs at a concentration of 500 nM each. Solutions were chilled at 4 °C for 15 min, followed by centrifugation at 12,000g for 10 min at 4 °C. 100 μL of supernatant was transferred to a clean NUNC 96-well plate and further diluted with 100 μL of H_2_O:MeOH (1:1), sealed with a preslit silicon mat, and stored at 4 °C until LC–MS analysis.

### LIVE/DEAD Staining to Assess Cell Death in MPS

MPS were treated with either vehicle (0.1% DMSO; *n* = 3), 1 μM polymyxin B (PMB, positive control; *n* = 2), 100 nM of C_12_-BAC (*n* = 2), or 100 nM C_14_-BAC (*n* = 3) for 1 week. A LIVE/DEAD staining viability/cytotoxicity kit containing calcein acetoxymethyl ester (AM) and ethidium homodimer-1 (EthD-1) was used, along with Hoechst 33342 (Thermo Fisher Scientific, Grand Island, NY), according to the manufacturer’s instructions. Briefly, calcein AM, EthD-1, and Hoechst 33342 were diluted in PBS (with Ca^2+^ and Mg^2+^) to final concentrations of 2 μM, 4 μM, and 50 μM, respectively. MPS chips were perfused through the luminal port for 20 min at 5 μL/min, followed by incubation at 37 °C for 10 min. After the staining procedure, chips were imaged using fluorescence microscopy to visualize live (green-stained) and dead (red-stained) cells, with Hoechst 33342 (blue-stained) as a nuclear marker. Sections of control, C_12_-BAC, and C_14_-BAC-treated tubules (*n* = 3) were imaged at 10× magnification. Within the images, randomly selected regions of interest (pixel area: 540 × 453; *n* = 3) were evaluated for quantitative analysis of cell viability (%). A cell counter in ImageJ (National Institutes of Health, Maryland, US) was used to manually count dead cells (red) and nuclei (blue).

Cell viability was calculated as

$$\mathrm{cell viability}=\frac{\#\mathrm{nuclei}-\#\mathrm{dead cells}}{\#\mathrm{nuclei}}\times 100$$

### Quantitation of Human Kidney Injury Molecule-1 (KIM-1) in Kidney MPS Effluents

MPS were treated with vehicle (0.1% DMSO; *n* = 2), 1 μM polymyxin B (positive control; *n* = 2), or 1 μM C_14_-BAC (*n* = 3) for 1 week. Chip effluents were collected every 24 h. The Human TIM-1/KIM-1/HAVCR Duoset ELISA kit (Bio-Techne, MN) was used to measure KIM-1 levels in the kidney MPS effluents following the manufacturer’s protocol.

### RNA Sequencing of Cells Extracted from MPS

PTECs derived from donor PT16 were cultured in kidney MPS and treated with a vehicle control (0.1% DMSO), C_12_-BAC, or C_14_-BAC (100 nM) for 1 week (*n* = 3 for each group). PTEC lysates were extracted from the MPS using 0.5 mL of RLT buffer (Qiagen). RNA was extracted using the RNeasy Micro Kit (Qiagen, 74004) and converted to cDNA with the SMART-Seq v4 Ultra-Low Input RNA Kit (Takara, 634891). Sequencing libraries were constructed using the SMARTer ThruPlex DNA-Seq Kit (Takara, R400676) and sequenced on a NovaSeq 6000 instrument (Illumina, San Diego, CA). FASTQ files were aligned to the Gencode GRCh38 v45 genome using HiSat2, and coordinates were sorted using samtools. Counts/gene were summarized using the Bioconductor Rsubread package using the Gencode v45 GTF file. Genes with consistently low expression were excluded, leaving 16,219 for analysis.^34^ The count data were then analyzed using the limma-voom pipeline in the Bioconductor edgeR package.^35^ Briefly, counts were normalized to library size by computing counts per million and then log-transformed to generate log counts per million (logCPM) values. The logCPM data are amenable to analysis using conventional linear regression, except for a dependence between the mean and variance. The relationship between the mean and variance was estimated using a locally weighted regression, which was then used to estimate observation-level weights. These weights are used in a weighted linear regression to adjust for the dependence between the mean and the variance. Multidimensional scaling (MDS) plots indicated some extra unobserved variability, which was adjusted for using a combination of sample weights and surrogate variables.^36^ Comparisons were made using empirical Bayes-adjusted contrasts, selecting genes using a false discovery rate (FDR) of <0.1, indicating we expect at most 10% false positives.^37^

### Targeted LC–MS/MS Analysis of BACs and Metabolites

BACs and BAC metabolites were quantified by ultra-performance liquid chromatography-tandem mass spectrometry (UPLC–MS/MS) using a Waters Synapt XS ion mobility Q-ToF mass spectrometer (Waters Corporation, Milford, MA) equipped with electrospray ionization (ESI) in positive mode [ESI­(+)] as previously described.^38,39^ Solvent gradients, analyte retention times (t_R_), and the precursor-to-product ion transitions monitored are as described in Supporting Information Tables S4 and S5.

### Sterol Analysis

PTECs derived from Donor PT16 were cultured and treated with vehicle (0.1% DMSO), 100 nM C_12_-BAC, or 100 nM C_14_-BAC for 48 h. Monolayers of PTECs were harvested, resuspended in 300 μL of 1X PBS, and then lysed by sonication in an ice bath for 30 min. Protein concentration was determined using the Bio-Rad DC protein mass assay (Bio-Rad, Hercules, CA). Ten μL of a sterol internal standard mixture was added to each sample, and lipid extraction was performed using the Folch method as described previously.^32^ Sterols were analyzed using ultra-performance liquid chromatography (UPLC) tandem mass spectrometry (MS/MS) on a Waters Xevo TQ-XS triple quadrupole mass spectrometer with atmospheric pressure chemical ionization (APCI) coupled to a Waters Acquity UPLC system (Waters Corporation, Milford, MA).

Sterols were separated by reverse-phase chromatography on a C_18_ column (1.7 μm, 2.1 × 100 mm, Phenomenex Kinetex) over 15 min using isocratic elution with 90% methanol containing 0.1% formic acid at a flow rate of 0.4 mL/min. Multiple reaction monitoring (MRM) was employed to track the dehydration of sterol and oxysterol [M + H]^+^ ions, resulting in the generation of [M+H–H_2_O]^+^ ions (Supplemental Tables S7 and S8 list the retention times and MS/MS transitions for the analytes and the standards used; Supplemental Table S9 describes the source and MS parameters). Data analysis was performed with TargetLynx (Waters Corporation, Milford, MA). Analyte concentrations in cells were determined relative to the internal standard levels and the relative response factor (RRF) of each analyte.

### Cell Cycle Analysis of 2D-Cultured PTECs Using Flow Cytometry

PTECs derived from donor PT21 (*n* = 3) were grown and treated with vehicle, 100 nM C_12_-BAC, or 100 nM C_14_-BAC in cell media for 1 week. The medium was changed every 24 h to mimic continuous dosing in an MPS. Known cell cycle inhibitors, nocodazole (Sigma-Aldrich; St. Louis, MO) at 200 nM and thymidine (Sigma-Aldrich; St. Louis, MO) at 2 mM,^40^ were used as positive controls after 24 h of treatment.

PTECs were fixed with 70% ethanol and stored at −20 °C until analysis. On the day of analysis, cells were resuspended in 1 mL of 3 μM 4′,6-diamidino-2-phenylindole (DAPI) dilactate (Thermo Fisher Scientific, Grand Island, NY) in staining buffer: 100 mM Tris (pH 7.4), 150 mM NaCl, 1 mM CaCl_2_, 0.5 mM MgCl_2_, and 0.1% Triton X-100. The cell suspension was incubated for 15 min at room temperature before undergoing flow analysis. Flow cytometry was performed on a BD Biosciences FACSymphony A3 Cell Analyzer. Forward and side-scatter voltages were adjusted to gain a sufficient cellular signal. 10,000 cellular events were collected, and cell cycle analysis was performed using FlowJo.

### Focal Adhesion Analysis of 2D Culture

PTECs from Donor WK4 were cultured and treated with vehicle control (0.1% DMSO), C_12_-BAC, or C_14_-BAC (100 nM). The cells were incubated at 37 °C and 5% CO_2_ for 1 week, with the media replaced every 24 h. The cells were prepared for a cell adhesion assay using CytoSelect 48-Well Cell Adhesion Assay (ECM Array, Colorimetric Format; Cell BioLabs, Inc.) according to the manufacturer’s instructions.

Briefly, cells were seeded at 300,000 cells/well and incubated at 37 °C with 5% CO_2_ for 2 h. Afterward, the media was discarded, and the cells were washed with PBS. PBS was aspirated, and the cell stain solution was added, followed by incubation at room temperature for 10 min. The stain solution was discarded, and the wells were washed with DI water, after which the plate was left to air-dry. An extraction solution was added to the wells, and the plate was incubated for 10 min on an orbital shaker. Absorbance at 560 nm was measured in a plate reader, and bovine serum albumin (BSA) was used as a negative control.

### Statistical Analyses

GraphPad Prism was used to perform various statistical analyses to determine the significance of the BAC effect on PTECs. IC_50_ ± standard error of the mean (SEM) is recorded for cell viability assays, determined by the inhibitor vs response (variable slope, four parameters) equation. All other data are reported as mean ± SD. Student’s *t*-test was used to compare two means, and analysis of variance (ANOVA) was used for multigroup comparison, followed by Dunnett’s test, to determine the significance of the BACs' effects in 2D and 3D culture (*, *P* < 0.05; **, *P* < 0.005; ***, *P* < 0.0005; ****, *P* < 0.00005).

## Results

### ω-OH Metabolites of BACs Are Much Less Cytotoxic Compared to Parent BACs in 2D Culture

We first examined the cytotoxicity of the individual parent BACs with varying alkyl chain lengths (C_12_-BAC, C_14_-BAC, C_16_-BAC) compared to the corresponding ω-OH BAC metabolites (ω-OH-C_12_-BAC, ω-OH-C_14_-BAC, and ω-OH-C_16_-BAC) in PTECs. We found that parent compounds displayed much greater cytotoxicity than their ω-OH metabolites (Figure 2; Table S2). C_12_-BAC, C_14_-BAC, and C_16_-BAC presented with IC_50_ values of 2.9, 1.5, and 1.3 μM, respectively. The ω-OH-C_12_-BAC metabolite did not exhibit obvious toxicity in the concentration range tested, whereas the IC_50_ of ω-OH-C_14_-BAC was estimated to exceed 10 μM. The IC_50_ value of ω-OH-C_16_-BAC, at 7.4 μM, is approximately 6-fold higher than that of the C_16_-BAC (Figure 2). These results suggest that CYP-mediated metabolism effectively mitigates the cytotoxicity of parent BACs (Figure 2).

**Figure 2:**
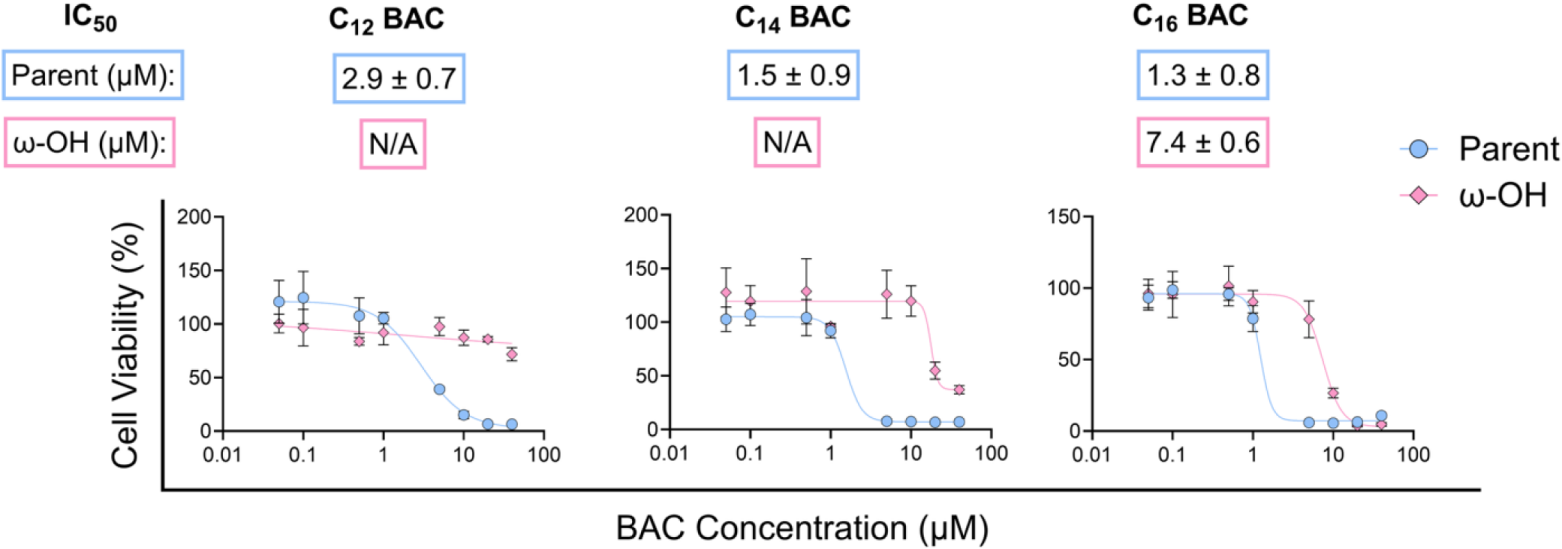
Cytotoxicity of C_12_-, C_14_-, and C_16_-BAC parent compared to the ω-OH BAC metabolite in 2D culture for 48 h. Parent BAC concentrations ranged from 0.4 to 40 μM. Data presented are IC_50_ ± SEM of parent BAC in both treatment groups. Dose–response curves were fitted by GraphPad Prism v. 10.6.

### Inhibition of CYP4F by HET0016 Does Not Significantly Enhance the Cytotoxicity of Parent BACs in 2D-Cultured PTECs

The potent CYP4F pan-inhibitor HET0016 was used to evaluate whether the toxicity of C_12_-BAC, C_14_-BAC, and C_16_-BAC to PTECs could be enhanced by blocking CYP4F-mediated metabolism. PTECs were treated with and without 0.5 μM HET0016, a sufficient concentration to inhibit CYP4F2 (IC_50_ = 125 nM)^26^ and CYP4F11 (IC_50_ = 49.5 nM).^41^ After 48 h, HET0016 was seen to have modestly enhanced C_12_-BAC toxicity, reducing the IC_50_ from 4.9 μM (without HET0016) to 2.4 μM (with HET0016), but had a negligible effect on C_14_-BAC and C_16_-BAC toxicity (Figure 3A; Table S3). We note that increased viability was observed at low C_14_-BAC concentrations in the absence of HET0016. We suggest that it may be due to either variability in plating the cells or stimulation of cell growth by low concentrations of C_14_-BAC. Slightly increased viability at low concentrations of BACs has been previously observed in a neuroblastoma cell line.^17^ Regardless, slightly higher viability at low and noncytotoxic BAC concentrations should not affect the IC_50_ value.

**Figure 3:**
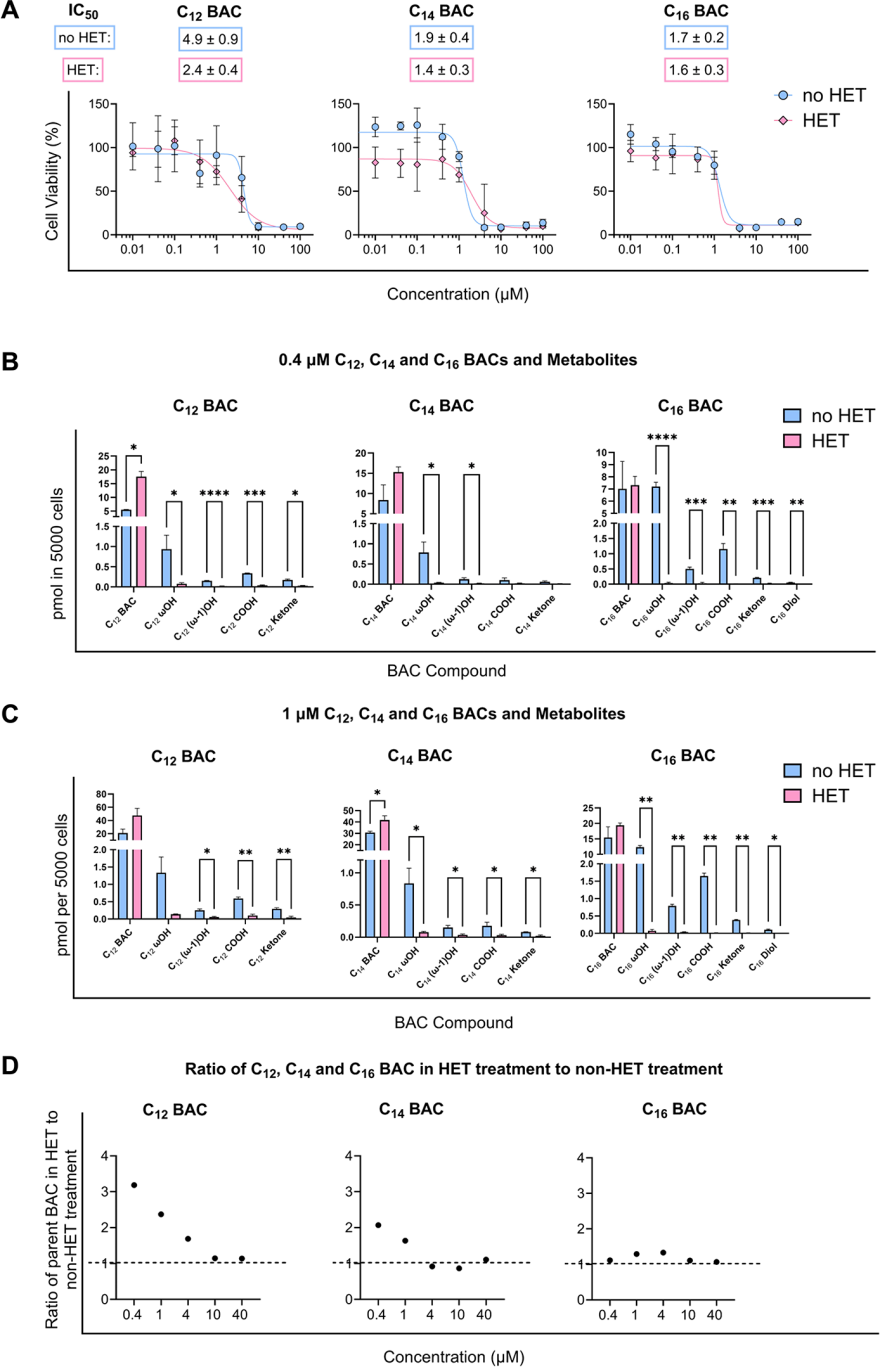
Cytotoxicity and metabolism of BACs in 2D-cultured PTECs in the presence or absence of CYP4F inhibitor, HET0016, for 48 h. (A) Cell viabilities of C_12_-, C_14_-, and C_16_-BAC-treated PTECs. Parent BAC concentrations ranged from 0 to 100 μM. Data is presented as IC_50_ ± SEM. Dose–response curves were fitted by GraphPad Prism v. 10.6. (B) Quantitation of parent BACs and BAC metabolites at 0.4 μM of BACs. (C) Quantitation of parent BACs and BAC metabolites at 1 μM of BACs. Statistical analysis is relative to control using Student’s *t*-test; *, *P* < 0.05; **, *P* < 0.005; ***, *P* < 0.0005; *n* = 3. (D) The average ratio of parent BAC concentration in HET-treated cells to non-HET Control cells treated with 0.4–40 μM of parent BACs.

We then quantified parent BACs and BAC metabolites in PTEC cell lysates using LC–MS/MS following treatment with C_12_-, C_14_-, or C_16_-BAC. HET0016-treated cells show potent inhibition of parent BAC metabolism across a range of BAC concentrations from 0.4 to 40 μM (Figure 3B and C showing 0.4 and 1 μM; Table S6). For example, at 1 μM, the ω-OH metabolites in non-HET0016-treated PTECs are on average 10, 11, and 154 times higher than those produced in HET0016-treated PTECs for C_12_-BAC, C_14_-BAC, and C_16_-BAC, respectively. In contrast, parent BAC levels show only slight differences between the two treatment groups. Plotting the ratio of parent BAC in the HET-treated groups to that in the non-HET-treated groups shows that the ratio approaches 1 as parent BAC concentrations increase (Figure 3D), indicating that the metabolic capacity of the cells is saturated at high parent BAC concentrations and is not susceptible to CYP4F inhibition. At lower parent BAC concentrations, however, HET0016 treatment slightly elevated parent BAC levels, particularly for C_12_-BAC. Thus, we observed a correspondence between the enhanced parent BAC levels precipitated by CYP4F inhibition and modestly enhanced cytotoxicity (e.g., C_12_-BAC). However, the magnitude of HET0016-induced enhancement of parent BAC accumulation and cytotoxicity was modest and was preferentially observed in C_12_-BAC-treated PTECs, with minimal impact on C_14_- and C_16_-BAC treatments.

### C_12_- and C_14_-BAC Significantly Increase Cell Death in PTECs Cultured in 3D Kidney MPS

We next examined the cytotoxicity of C_12_- and C_14_-BAC at 100 nM, a physiologically relevant concentration based on the quantified levels of parent BACs in human blood (median level of 2.5 ng/mL and max level of 68 ng/mL, equivalent to 7 and 180 nM, respectively),^6^ in the kidney-on-a-chip MPS over 7 days of exposure, in comparison with a negative control (0.1% DMSO) and a positive control [1 μM polymyxin B (PMB)] (Figure 4A). Based on a previous study in rats,^20^ BAC levels in the kidney are approximately 15 times those in the blood 24 h after oral administration, which justifies using 100 nM (∼15 times the median concentration in human blood) in the MPS. Increasing dead cells are visible in tubules treated with longer parent BAC chain length compared to the control tubules (Figure 4B). Analysis of live/dead cells relative to total nuclei corroborates that the extent of cell death is dependent on parent BAC chain length (Figure 4C), with the longer C_14_-BAC being more toxic.

**Figure 4:**
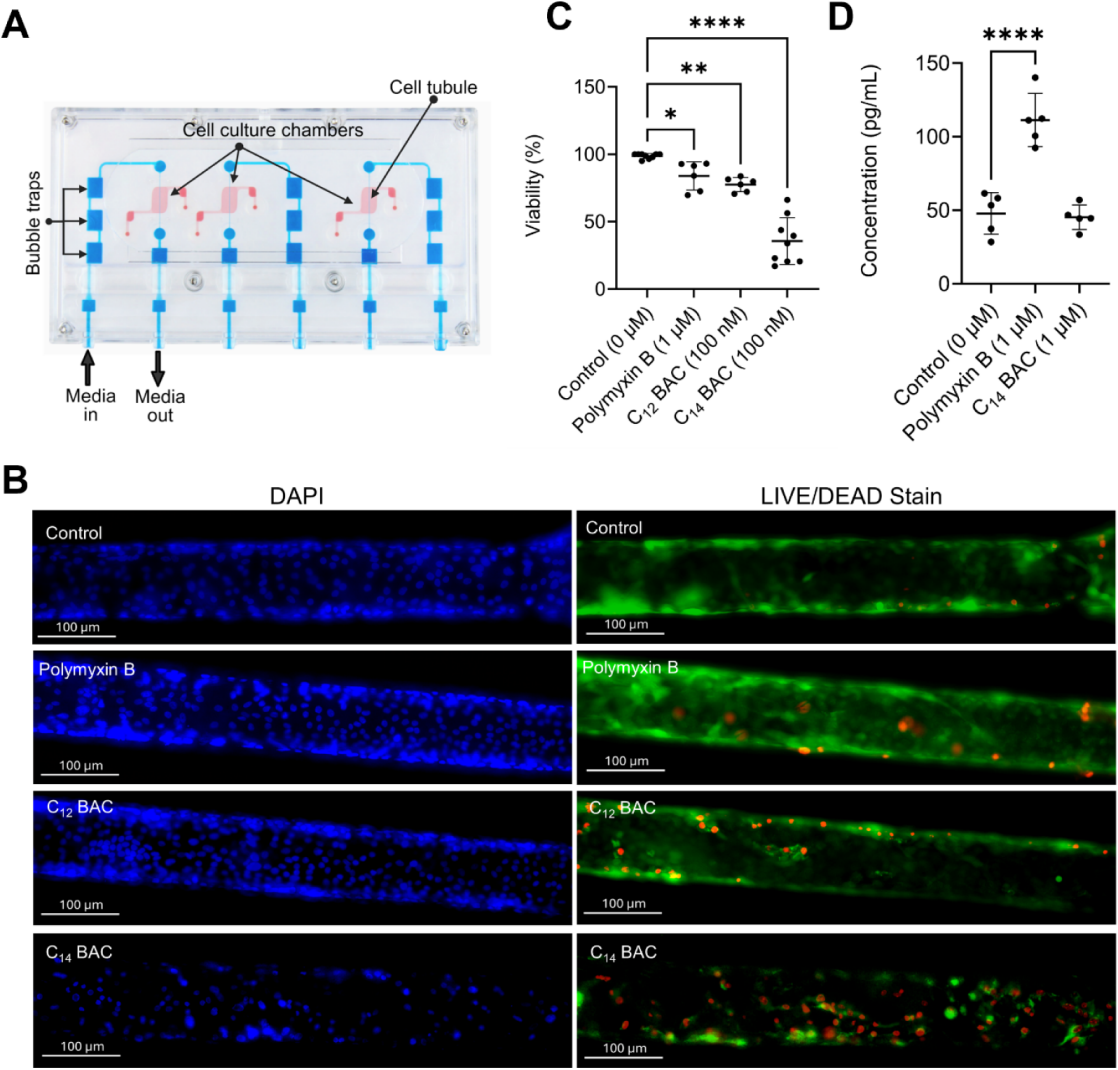
Characterization of kidney injury in C_12_ and C_14_ BAC-treated MPS for 1 week. (A) Nordis Triplex chip, ©Nortis, Inc. (B) DAPI and LIVE/DEAD staining images (magnification: 10×) of control (*n* = 3), C_12_-BAC (*n* = 2), C_14_-BAC (*n* = 3), and polymyxin B (positive control; *n* = 2). (C) Quantitative analysis of cell viability (%) in treatments using a cell counter in ImageJ (National Institute of Health, Maryland, US). Regions of interest (pixel area: 540 × 453; *n* = 3 for each tubule image) were randomly chosen across all biological replicates of each treatment. Dead (red) cells were manually counted, and viability was calculated compared to the number of DAPI-stained cells. (D) Concentrations of KIM-1 released in MPS effluents of control (*n* = 2), polymyxin B (1 μM; *n* = 2), and C_14_-BAC (1 μM; *n* = 3) treatments over days 1–5. Statistical analysis was performed using one-way ANOVA, followed by posthoc analysis relative to the Control. Values are reported as the mean ± SD (*, *P* < 0.05; **, *P* < 0.005; ****, *P* < 0.00005).

### Kidney Injury Biomarker, KIM-1, in Kidney MPS Effluents Is Not Affected by Parent BAC Treatment

Kidney injury molecule-1 (KIM-1) is a transmembrane glycoprotein that is cleaved at the plasma membrane and is a sensitive marker of acute kidney injury.^42^ The level of released KIM-1 ectodomain was assessed in the effluents of MPS treated with 1 μM of C_14_-BAC, 1 μM of PMB (positive control),^31^ or vehicle (0.1% DMSO) control for 7 days. We found that PMB indeed led to a significantly increased release of KIM-1 over the first 5 days, but C_14_-BAC treatment did not induce a significant release of KIM-1 in the effluents (Figure 4D).

### Quantitation of Parent BAC and Metabolites in Kidney MPS Effluents and 2D-Cultured PTECs

To assess BAC metabolism and disposition in the 3D MPS, the levels of BAC and BAC metabolites were quantified in effluents of MPS treated with 100 nM C_12_- or C_14_-BAC (*n* = 3 for each group). Parent C_12_-BAC was not detectable in the effluents in C_12_-BAC-treated MPS. However, C_12_-BAC metabolites, including the ω-OH, (ω-1)-OH, and COOH, were detected in effluents at low nM levels after about 24 h, with the ω-OH being the primary excreted metabolite (Figure 5A). The secondary metabolites, (ω-1)-ketone and (ω, ω-1)-diol, were not detected. Furthermore, C_14_-BAC or C_14_-BAC metabolites were not observed in the effluents of the C_14_-BAC-treated MPS.

**Figure 5:**
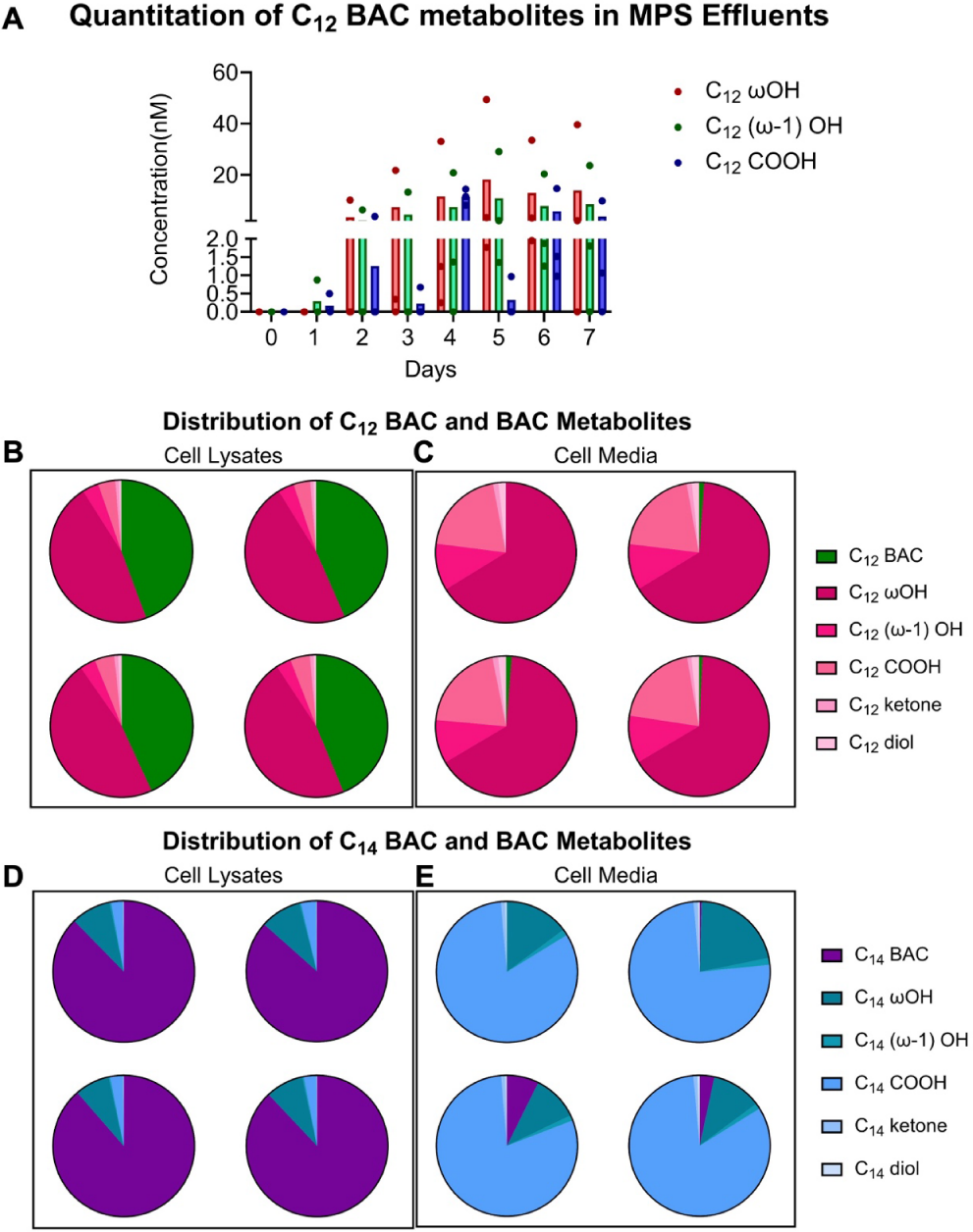
BAC parent and metabolite distributions in 2D- (B–E) and 3D-cultured PTECs (A). PTECs were treated with 100 nM C_12_- or C_14_-BAC for 48 h in 2D PTECs and 1 week in 3D PTECs. (A) Quantitation of C_12_ metabolites in effluents of 3D MPS (*n* = 3 for each group). C_12_-BAC, C_14_-BAC, and C_14_-BAC metabolites were not detected in the 3D effluents. (B) Quantitation of C_12_-BAC and metabolites in 2D-culture cell lysates. (C) Quantitation of C_12_-BAC and metabolites in 2D culture media. (D) Quantitation of C_14_-BAC and metabolites in 2D-culture cell lysates. (E) Quantitation of C_14_-BAC and metabolites in 2D-culture media.

To further examine the metabolism and accumulation of parent BACs and metabolites in PTECs, parallel experiments were carried out in 2D-cultured PTECs treated with vehicle control, 100 nM C_12_-BAC, or 100 nM C_14_-BAC for 48 h (*n* = 4) (Figure 5B–E). Parent C_12_-BAC was detected at subpmol levels in the cell media (mean = 0.1 pmol/5,000 cells), but at a 228-fold higher level in the cell lysate (mean = 22.8 pmol/5,000 cells), indicating significant intracellular accumulation of the parent BAC (Figure 5B–C). On the other hand, C_14_-BAC was detected at a 423-fold higher level in the cell lysates (mean = 42.3 pmol/5000 cells) relative to the media (mean = 0.1 pmol/5000 cells) (Figure 5D-E).

The metabolites ω-OH-C_12_-BAC and ω-OH-C_14_-BAC were detected at the highest levels in the cell lysates, with mean amounts of 73.5 pmol/5,000 cells and 11.6 pmol/5,000 cells, respectively (Figure 5B and D). The intracellular mean amounts of (ω-1)-OH-C_12_-BAC and COOH-C_12_-BAC were 5.6 pmol/5,000 cells and 6.5 pmol/5,000 cells, respectively, while those of (ω-1)-OH-C_14_-BAC and COOH-C_14_-BAC were lower, at 0.4 pmol/5,000 cells and 4.0 pmol/5,000 cells, respectively. Minor metabolites, including (ω-1)-ketone-C_12_-BAC, C_12_-BAC-(ω,ω-1)-diol, (ω-1)-ketone-C_14_-BAC, and C_14_-BAC-(ω,ω-1)-diol, were detected at lower levels (mean ≤ 1 pmol/5,000 cells).

In the extracellular media, ω-OH-C_12_-BAC was the predominant metabolite (mean = 10.2 pmol/5,000 cells), followed closely by COOH-C_14_-BAC (mean = 6.5 pmol/5,000 cells) (Figure 5C). Other metabolites, including COOH-C_12_-BAC (mean = 5.0 pmol/5,000 cells), (ω-1)-OH-C_12_-BAC (mean = 1.2 pmol/5,000 cells), and (ω-1)-ketone-C_12_-BAC (mean = 2.0 pmol/5,000 cells), were also detected. The ω-OH-C_14_-BAC, (ω-1)-OH-C_14_-BAC, (ω-1)-ketone-C_14_-BAC, and both diol metabolites were present at lower levels (mean ≤ 1 pmol/5,000 cells). Collectively, these findings indicate that parent BACs preferentially accumulate intracellularly, whereas their metabolites are more efficiently excreted into the extracellular media.

### Transcriptomic Analysis of PTECs in the Kidney MPS Treated with Individual BACs

Transcriptional changes were assessed using RNA sequencing of PTECs (derived from donor PT16) in the kidney MPS after 1-week exposure to vehicle control, 100 nM C_12_-BAC, or 100 nM C_14_-BAC. The multidimensional scaling (MDS) plot shows clear separation between the parent BAC-treated groups and the control group (Figure 6A). C_12_- and C_14_-BAC identified 31 and 1767 differentially expressed genes, respectively (FDR = 0.1), with a greater number of downregulated genes than upregulated genes (Figure 6B). C_12_-BAC altered 14 biological pathways, whereas C_14_-BAC altered 49 pathways, with 7 pathways being shared by both treatments (*P* ≤ 0.05) (Figure 6C). The top 10 differentially affected pathways, along with their respective genes, are shown in Supplemental Tables S10 and S11. All observed genes, including nonsignificant genes, are listed in Table S16 as an Excel file.

**Figure 6:**
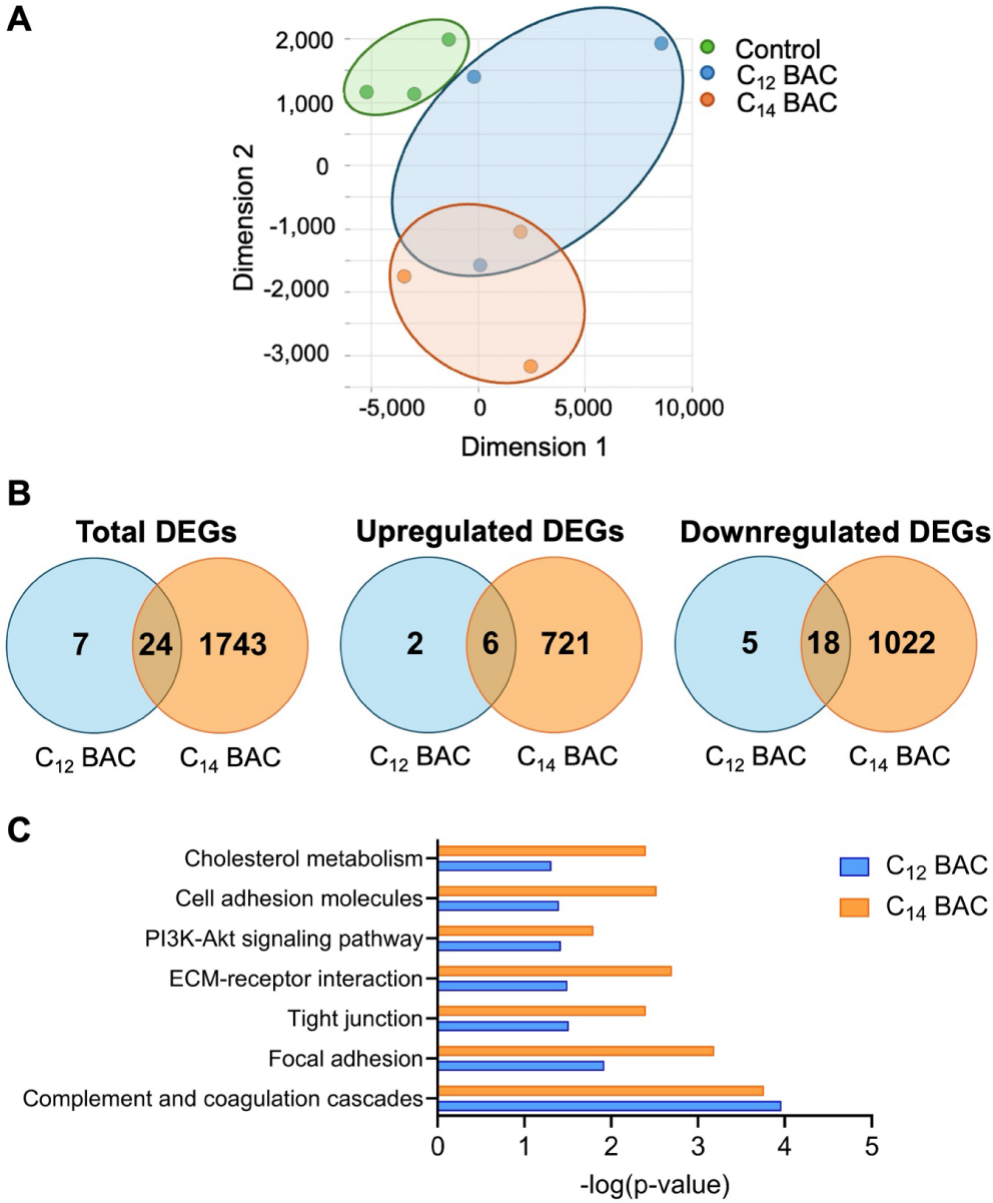
Transcriptional response in MPS to C_12_- or C_14_-BAC exposure at 100 nM for 1 week. (A) MDS plot of transcriptional changes in vehicle, C_12_-BAC, and C_14_-BAC-treated MPS. (B) Comparison of differentially expressed genes (DEGs) in C_12_-BAC and C_14_-BAC treatments. (C) Overlapping differentially expressed pathways between C_12_- and C_14_-BAC treatments, expressed as -log­(*p*-value). *N* = 3 for each group.

The top shared pathways include focal adhesion, extracellular matrix (ECM)-receptor interaction, cell adhesion molecules, and a tight junction. C_14_-BAC affected a greater number of genes related to the aforementioned pathways than did C_12_-BAC. Furthermore, C_14_-BAC affected a greater number of genes in the PI3K-Akt signaling pathway (Figure 6C).

Notably, previous work has demonstrated that parent BACs significantly affect genes involved in cholesterol homeostasis and biosynthesis in mouse neuroblastoma cells.^17^ Although there was no overlap in the genes involved in cholesterol homeostasis between C_12_- and C_14_-BAC, C_12_-BAC-treated MPS showed decreased expression of *ABCA1*, a cholesterol-efflux transporter, while C_14_-BAC-treated MPS showed increased expression of *HMGCR*, the rate-determining step of cholesterol biosynthesis, similar to patterns seen previously.^17^

### Sterolomics of 2D-Cultured PTEC Cell Lysates

Previous studies have shown that parent BACs of different chain lengths can alter cholesterol biosynthesis in neuronal cell lines and in mouse neonatal brains.^17,18^ Cholesterol metabolism was a significantly altered pathway in the transcriptomics analysis of human PTECs isolated from MPS experiments (C_12_BAC: *P* = 0.049; C_14_BAC: *P* = 0.004). To validate transcriptomic changes in cholesterol metabolism, we performed sterolomic analysis of 2D-cultured PTECs treated with 100 nM C_12_- or C_14_-BAC for 48 h. We found that both treatments led to significantly increased levels of 7-dehydrocholesterol (7-DHC) and 7-dehydrodesmosterol (7-DHD), indicating that parent BAC treatment may inhibit DHCR7 as observed in neuronal cell lines previously.^17^ C_12_-BAC treatment led to greater accumulation of 7-DHC and 7-DHD, indicating that it is a more potent inhibitor than C_14_-BAC (Figure 7). We also observed a decrease in the levels of 8-dehydrocholesterol (8-DHC) and an increase in the levels of zymosterol, similar to those reported by Herron et al.^17^ Despite altered levels of cholesterol precursors, the levels of cholesterol and total sterols did not change significantly, although there was a trend toward a decrease for cholesterol (*P* = 0.10) in the C_12_-BAC-treated group (Figure 7).

**Figure 7:**
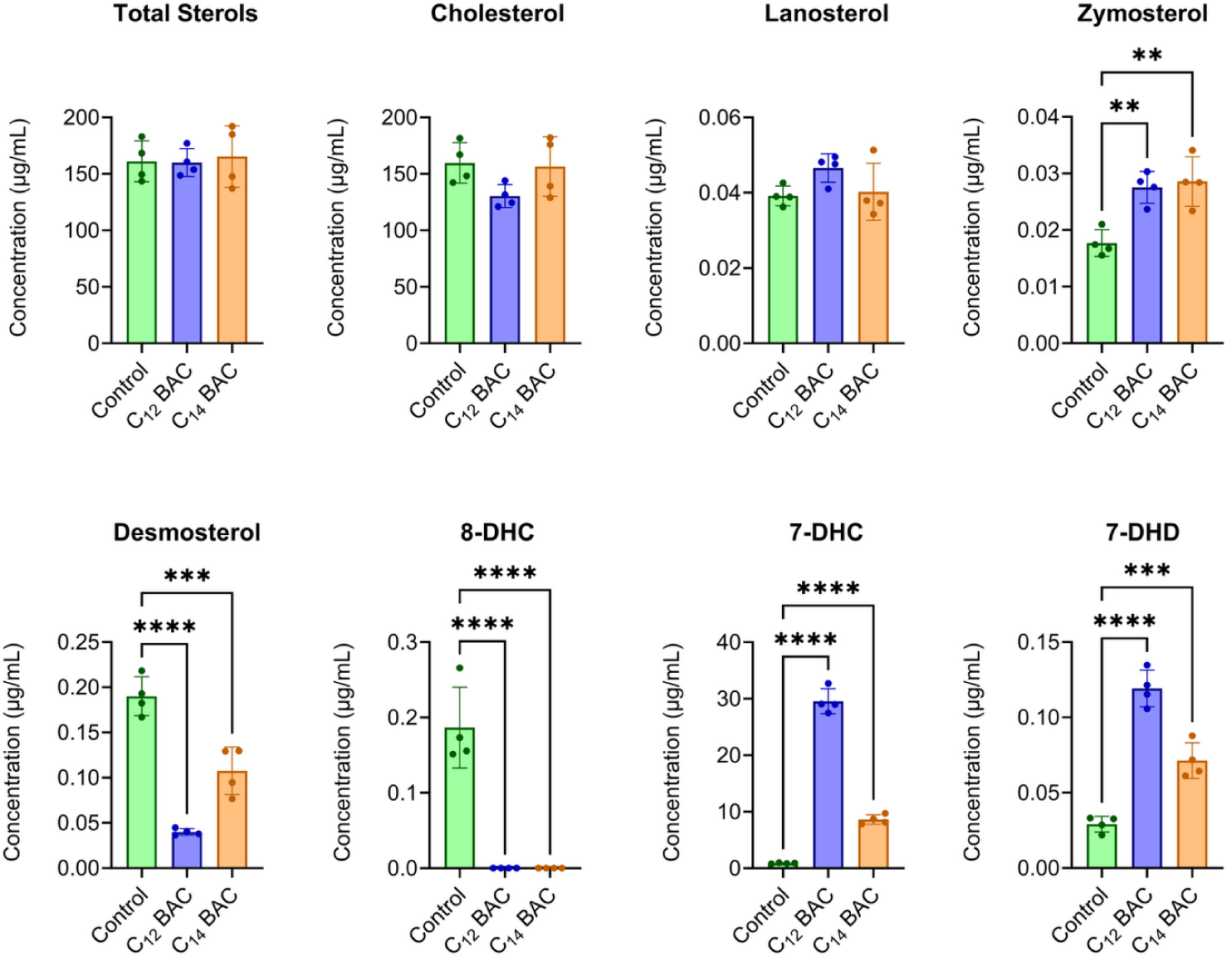
Quantitation of sterols in 2D-cultured PTECs exposed to individual BACs for 48 h. Sterols were quantified in cell lysates after exposure to C_12_- or C_14_-BAC (100 nM) in 2D culture for 48 h. Statistical analysis was performed using one-way ANOVA followed by posthoc analysis compared to the Control. Values are reported as the mean ± SD (*, *P* < 0.05; **, *P* < 0.005; ***, *P* < 0.0005; ****, *P* < 0.00005).

### C_12_ and C_14_-BAC Significantly Reduce Cell Adhesion to the Extracellular Matrix (ECM) in 2D Cultures

Because RNA sequencing revealed that many of the shared pathways in C_12_- and C_14_-BAC-treated chips involved cellular/focal adhesion and ECM-receptor interaction, we investigated the adhesion of 2D-cultured PTECs to various ECM proteins after chronic exposure to low levels of individual BACs. Of the five adhesion proteins assessed, both C_12_- and C_14_-BAC treatments led to decreased adhesion of laminin I (Figure 8A–B). C_14_-BAC additionally shows a decrease in adhesion to collagen I and fibrinogen (Figure 8B).

**Figure 8:**
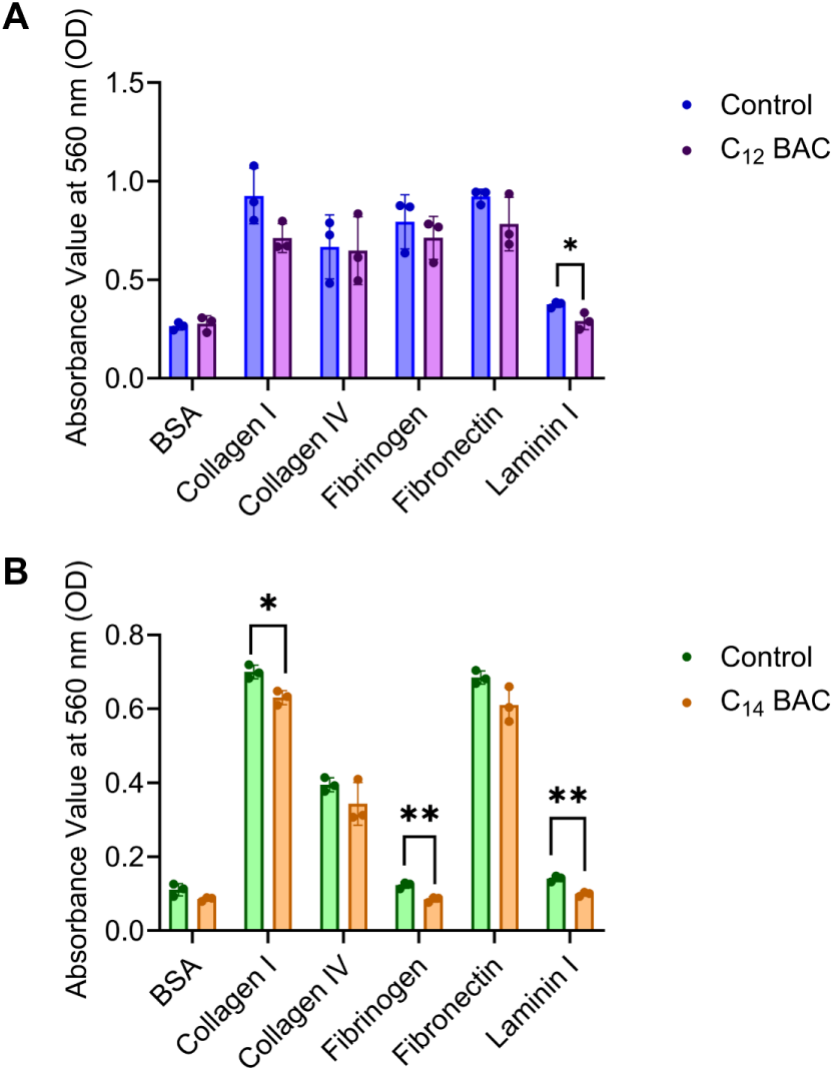
Cell adhesion was assessed in 2D-cultured PTECs exposed to (A) C_12_-BAC or (B) C_14_-BAC at 100 nM for 1 week. Absorbance was measured at 560 nm in a single extracellular matrix protein. *N* = 3 for each group. Statistical analysis was performed using one-way ANOVA followed by posthoc analysis compared to the Control. Values are reported as the mean ± SD (*, *P* < 0.05; **, *P* < 0.005).

### Parent BAC Treatments Significantly Affect the Cell Cycle in 2D Culture

Because focal adhesion can affect the cell cycle, and the cell cycle was identified as a significantly affected pathway by C_14_-BAC in transcriptomic analysis (*P* = 0.003), we performed cell cycle analysis of PTECs treated with 100 nM of C_12_- or C_14_-BAC for 1 week using flow cytometry. As shown in Figure 9 and Supporting Information Table S12, we observed a significant increase in S-phase arrest in both C_12_- and C_14_-BAC-treated groups compared with vehicle and positive controls (Supporting Information Table S13 and Figure S1), while decreases in the G1 phase accompanied these changes.

**Figure 9:**
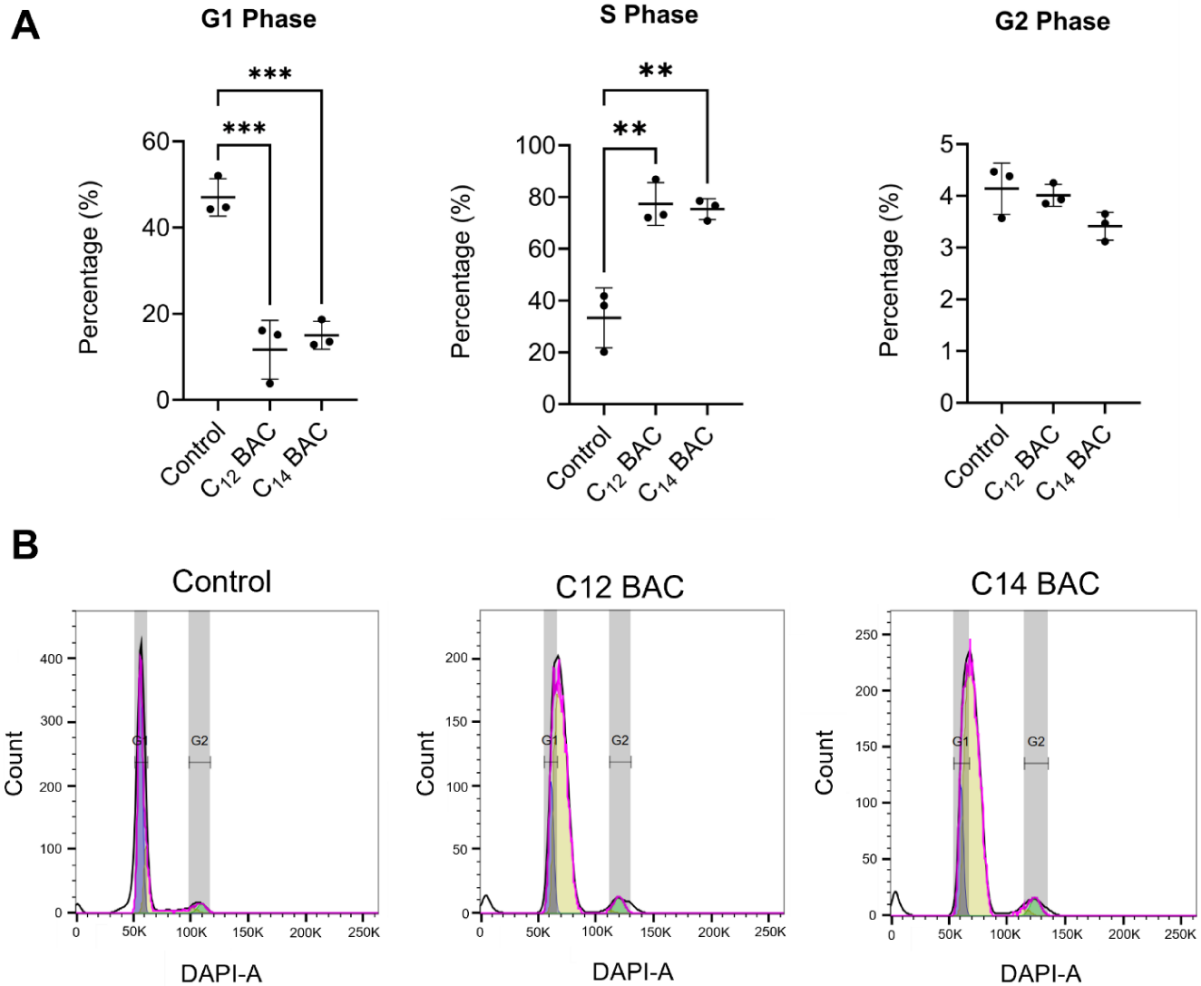
Cell cycle distribution of 2D-cultured PTECs exposed to C_12_- or C_14_-BAC (100 nM) for 1 week. (A) Percentage distribution of cell cycle phases G1, S, and G2 in C_12_- or C_14_-BAC-exposed 2D-cultured PTECs. (B) Example flow cytometry graph of each treatment. Statistical analysis was performed using one-way ANOVA followed by post hoc analysis compared to the Control. Values are reported as the mean ± SD (*, *P* < 0.05; **, *P* < 0.005). *N* = 3 for each group.

## Discussion

BAC metabolism by CYP enzymes has been previously characterized, with ω-OH metabolites as the primary metabolites^24^ (Figure 1). However, the toxicity of parent BACs and BAC metabolites toward kidney cells had not been assessed prior to this study. In 2D-cultured human PTECs, ω-OH metabolites of C_12_-, C_14_-, and C_16_-BAC displayed significantly lower toxicity than their associated parent, suggesting that an appropriate rate of BAC metabolic clearance may detoxify the parent compounds in the kidney (Figure 2). Thus, we tested whether the CYP4F inhibitor HET0016 could block parent BAC metabolism and exacerbate the toxicity of parent BACs. However, while HET0016 effectively blocked metabolism, it did not significantly raise the exposure levels of the parent BACs, suggesting that PTECs’ basal metabolic capability is insufficient to detoxify BACs (Figure 3).

Although the parent BACs showed minimal toxicity at 100 nM in 2D culture, they exhibited clear toxicity in 3D-cultured PTECs in the “kidney-on-a-chip” MPS, as indicated by an increased proportion of dead cells (Figure 4). This is likely because the 3D MPS more accurately represents the human proximal tubule structure and functions, enabling the simulation of longer exposure times that may not be achievable in 2D culture. Interestingly, we observed only the metabolites of C_12_-BAC in the MPS effluents, but not the parent C_12_- or C_14_-BACs or the metabolites of C_14_-BAC (Figure 5). The absence of parent BACs in the effluent could indicate that they accumulate in the cells. However, it is also possible that a significant fraction of these compounds could bind to the plastic materials in the kidney chip. Parent BACs are known to bind to plastic surfaces in well plates, significantly reducing the free concentration available for cellular uptake.^22^ This phenomenon is highly dependent on concentration and chain length, where plastic sorption positively correlates with increasing chain length. In addition, the authors observed saturation of plastic surfaces of wells at higher medium concentrations, suggesting that a low concentration of 100 nM may be prone to substantial sorption onto plastic. However, although initially free parent BAC exposure may experience some degree of adsorption to the plastic material, the kidney chip operates under continuous perfusion, and any initial adsorption to the device would reach saturation,^22^ limiting its impact on steady-state exposure during chronic dosing. Nevertheless, despite the lack of detection of parent BACs in the effluent, significant cytotoxicity caused by C_12_- and C_14_-BACs was observed at this low, physiologically relevant concentration (Figure 4).

A parallel 2D experiment revealed that parent C_12_- and C_14_-BAC indeed accumulate intracellularly, consistent with previous animal studies on kidney accumulation, and provides a rationale for the lack of parent BACs in 3D effluents. Our recent study using cells overexpressing human organic cation transporters (hOCTs) and multidrug and toxin extrusion proteins (hMATEs) found that C8- and C_10_-BACs are substrates of these transporters, whereas long-chain parent BACs do not rely on them for uptake, likely due to their high lipophilicity.^39^ hOCT2 and hMATE1/2K are the major isoforms expressed in the basal and apical membranes of renal proximal tubule cells, respectively.^39,43^ Using a hOCT2/hMATE1-expressing cell line as a renal excretion model, we found that parent BACs indeed accumulated significantly intracellularly.

Though toxicity by C_14_-BAC at 100 nM was observed with LIVE/DEAD staining, the level of the kidney injury marker, KIM-1,^44–46^ in the effluents did not significantly increase even with the treatment of 1 μM C_14_-BAC for 1 week relative to controls (Figure 4D).^31^ This absence of a significant increase suggests that significant release of KIM-1 would be unlikely at the lower 100 nM concentration used in the remaining experiments. Such observation is not uncommon for nephrotoxins. For example, KIM-1 was also not significantly affected by the nephrotoxin ochratoxin A (OTA), despite OTA potently inducing cell death, as revealed by LIVE/DEAD staining.^33^ In the context of BAC exposure, the absence of KIM-1 release may reflect a unique mechanism of toxicity that is not strongly associated with upregulation of kidney injury biomarkers. A lack of KIM-1 release has also been reported for other nephrotoxins^33,47^ such as ochratoxin A. We thus aimed to identify alternative mechanisms of parent BAC toxicity using transcriptomic analysis of the PTECs cultured in MPS, which revealed significantly affected pathways by both C_12_- and C_14_-BACs, including cholesterol metabolism, adhesion-related pathways (focal adhesion, ECM-receptor interaction, and cell adhesion molecules), the PI3K-Akt signaling pathway, and the complement and coagulation cascade pathway (Figure 6).

Analysis of C_14_-BAC-treated PTECs in 3D MPS revealed 41 DEGs out of 240 genes in the PI3K-Akt signaling pathway (*P* = 0.016), including downregulation of the mitogen-activated protein kinase kinase 1 (MEK/MAP2K1) (log_2_FC = −0.385, *P* = 0.044). MEK is an upstream regulator of ERK, which differentially regulates the constitutive KIM-1 shedding. Inhibition of MEK significantly blocks the constitutive shedding of the KIM-1 ectodomain in 769-P cells, which could account for the lack of KIM-1 release in C_14_-BAC-treated PTECs.^48^

We carried out sterolomics to validate the altered cholesterol metabolism pathway, revealing that 100 nM C_12_- or C_14_-BAC significantly inhibits cholesterol biosynthesis at the step of DHCR7 in 2D-cultured PTECs, consistent with our previous work (Figure 7).^17^ However, we acknowledge that inhibition of cholesterol biosynthesis may not be the underlying cause of the apparent cell death because C_12_-BAC was a more potent DHCR7 inhibitor, whereas C_14_-BAC induced greater cell death in PTECs (Figure 4C). The degree of DHCR7 inhibition does not correlate with cytotoxicity, and total cholesterol levels remained unchanged despite changes in sterol precursors. While disruption of cholesterol biosynthesis can influence proximal tubular function through effects on membrane composition, transporter activity, and cellular stress responses, the extent to which this contributes to the observed cytotoxicity appears to be small. This suggests that alterations in other biochemical pathways, such as focal adhesion and the cell cycle, may contribute more directly to PTEC injury. However, because of the role of cholesterol biosynthesis in cell proliferation and differentiation,^49^ inhibiting cholesterol biosynthesis could affect the repair of the proximal tubule.

The altered focal adhesion and ECM-related pathways suggest that parent BACs may decrease specific cellular-ECM protein interactions, which are crucial for regulating a range of significant cellular functions, including cell signaling complexes, migration, and survival. Indeed, C_14_-BAC-treated PTECs showed reduced binding to collagen I, laminin I, and fibrinogen, which are encoded by the *COL1A1*, *LAMA1*, and *FGB* genes, respectively. Although C_14_-BAC did not lead to significant changes in *COL1A1* and *LAMA1*, it led to a significant downregulation of the *FGB* gene (log_2_FC = −1.673, *P* = 0.006), which encodes the fibrinogen beta chain. While fibrinogen is a crucial component of cell adhesion and spreading, it is also significantly involved in blood coagulation processes. Defects in the production of hemostatic proteins, including fibrinogen, are associated with bleeding disorders.^50,51^ Interestingly, the complement and coagulation cascade was a significantly altered pathway in our RNA-seq data (*P* = 1.744e^–4^). C_12_-BAC was additionally found to induce reduced binding to laminin I, though no significant changes in the *LAMA1* gene were observed. Additional studies are needed to elucidate the mechanisms that govern the decreased adhesion of parent BAC-treated cells.

Focal adhesion can affect cell cycle progression.^52^ Indeed, the cell cycle pathway was significantly affected by C_14_-BAC in the 3D MPS but not by C_12_-BAC. However, flow cytometry revealed that both C_12_- and C_14_-BAC treatments arrested cells in the S phase of the cell cycle in the 2D culture (Figure 9). RNA sequencing also highlights the ERBB2 gene (also known as HER2), which is significantly downregulated in both C_12_-BAC- and C_14_-BAC-treated MPSs (C_12_-BAC: Log_2_FC = −0.401, *P* = 0.041; C_14_-BAC: Log_2_FC = −0.0530, *P* = 0.003). ERBB2 receptor expression is widely distributed in the kidney, with primary expression in the proximal tubule. This receptor signaling mediates a variety of renal physiological responses, including cell proliferation.^53^ Low ERBB2 receptor expression in the kidney is linked to multiple types of kidney cancer, such as renal clear cell carcinoma (ccRCC). Specifically, low ERBB2 expression has been shown to promote ccRCC tumorigenesis by regulating the cell cycle.^54^

This study has several limitations. First, while the kidney-on-a-chip MPS more closely mimics the structure and function of the human proximal tubule than 2D-cultured cells, the small number of cells prevents us from validating transcriptomic changes in the MPS system, such as focal adhesion, cell cycle, and cholesterol biosynthesis. Therefore, these phenotypes were validated in 2D-cultured cells instead. Structural and functional differences between 2D and 3D culture systems may influence the magnitude and dynamics of pathway activation, but the overlap in activation of major pathways supports their biological relevance to parent BAC-induced injury. However, we acknowledge that the cross-platform comparison is inferential and that the proposed toxicity mechanisms were not directly validated in the MPS system. Second, interdonor variability may contribute to quantitative differences in BAC metabolism and toxicity observed across experiments. However, our previous publication^30^ found that the coefficient of variation in metabolic functions toward vitamin D and its derivatives among three different donors is not drastic, within 30% (see Tables S14 and S15 for data extracted from the original characterization of the kidney MPS by Weber et al.). In another study of polymyxin-B (PMB) nephrotoxicity in the kidney MPS, transcriptional responses of 6 different donors to PMB were similar, where they concluded that “dominant transcriptional differences were in response to drug treatment rather than inter- and intra-individual donor response variation”.^31^ In addition, while donor-specific differences may influence the magnitude of responses, we show consistent directionality of cytotoxic effects and pathway perturbations. Nonetheless, future studies incorporating additional donors will be important to assess the generalizability of these responses. Third, future studies are necessary to investigate the molecular mechanisms underlying alterations in cell-ECM interactions and the cell cycle.

In summary, this study used a combination of conventional 2D cell culture and novel 3D MPS to assess nephrotoxicity by a class of widely used antiseptic compounds, BACs, which revealed potent toxicity of parent BACs, lack of BAC metabolism, significant intracellular accumulation of parent BACs in the cells, and significant alterations in cholesterol biosynthesis, focal adhesion, and the cell cycle. Findings from this study suggest that chronic exposure to low levels of parent BACs may adversely affect kidney health.

## Supplementary Material





## Notes

PTEC donor demographics; data for Figures 2, 3a, 3b, and 3c; LC–MS solvent gradients, MS/MS transitions, and retention times for BACs and their metabolites; retention times, MS/MS transitions, and source conditions for sterolomics; top 10 differentially expressed pathways in BAC-treated MPS; top 10 differentially expressed genes in BAC-treated MPS; data for cell cycle distribution from flow cytometry analysis (PDF)
All observed genes and their fold changes and adjusted *p*-values from the RNA sequencing analysis (Table S16) (XLSX)
