# Supplementary material for: Benzalkonium Chloride-Induced Nephrotoxicity in 2D Cultures and a Human Kidney-on-a-Chip System
Source: Environ Sci Technol. 2026 May 25;60(22):15557–69. doi: 10.1021/acs.est.5c15625 (PMC13262029; doi:10.1021/acs.est.5c15625)
Supplement: Supplementary file 1 [file es5c15625_si_001.pdf]

Supporting Information

Benzalkonium Chloride-Induced Nephrotoxicity in 2D Cultures and a Human Kidney-on-a-Chip System

Marie N. Brzoska[1], Ryan P. Seguin[1], James W. MacDonald[3], Jade Yang[1], Vanessa A. Lopez[1], Sydney Arnzen[1], Edward J. Kelly[2,3], Libin Xu[1,3]

[1]Department of Medicinal Chemistry, University of Washington, Seattle, WA 98195
[2]Department of Pharmaceutics, University of Washington, Seattle, WA 98195
[3]Department of Environmental and Occupational Health Sciences, University of Washington, Seattle, WA, 98195

*Correspondence:
Libin Xu, Ph.D.
Email: libinxu@uw.edu
Tel: (206) 543-1080

Table of Contents

Table S1. PTEC Donor Demographics. 3

Tables S2. Cell viability (means ± SEM) of 2D-cultured PTECs in the presence of C12-, C14-, and C16-BAC parent compared to the ω-OH BAC metabolite over 48 hours (for Figure 2). 4

Tables S3. Cell viability (means ± SEM) of 2D-cultured PTECs treated with different concentrations of BACs in the presence or absence of CYP4F inhibitor, HET0016, for 48 hours (for Figure 3a). 5

Table S4. Solvent gradients of LC-MS targeted analysis of BACs and metabolites. 6

Table S5. Precursor ion transitions and analyte retention times of parent BACs and metabolites[1]. 6

Table S6. Parent BAC and metabolite for C_{12}, C_{14} and C_{16} BAC in Figure 3b and 3c (values are in pmol per 5000 cells; SD = standard deviation). 7

Table S7. Sterol retention times, MS/MS transitions, and concentrations. 8

Table S8. Retention times and MS/MS transitions for sterol internal standards. 8

Table S9. Source and MS conditions for sterolomics. 9

Table S10. Top 10 differentially expressed pathways in C_{12}- or C_{14}-BAC-treated MPS. 10

Table S11. Top 10 differentially expressed genes in C12 and C14 BAC-treated MPS. 11

Table S12. Cell cycle distribution from flow cytometry analysis of BAC treatments. 12

Table S13. Cell cycle distribution from flow cytometry of positive controls. 12

Figure S1. Cell cycle distribution from flow cytometry analysis of positive controls. N = 3 per group. *, $P < 0.05$; **, $P < 0.005$; ***, $P < 0.0005$; ****, $P < 0.00005$ 13

Tables S14. Quantitative data for Figure 7A in Weber et al., *Kidney Int*. 2016, 90, 627: 1,25-$(OH)_2$-Vitamin D_3, 4β,25−$(OH)_2$-Vitamin D3, 24,25-$(OH)_2$-Vitamin D_3 means, SEM, SD, and coefficient of variations (CV). 14

Tables S15. Quantitative data for Figure 7B in Weber et al., *Kidney Int*. 2016, 90, 627: Calcidiol and calcidiol+calcitriol SEM, SD, and coefficient of variations (CV). 14

Table S1. PTEC Donor Demographics.

Donor	Age	Sex	Race/Ethnicity	Cause of Death	Pre-existing Conditions
PT16	59 years	Male	Caucasian	Head trauma/blunt injury	None
PT11	64 years	Male	Caucasian	Cerebrovascular stroke	Diabetes, hypertension, coronary artery disease, gastrointestinal disease
PT13	36 years	Male	Caucasian	Anoxia	None
PT21	52 years	Female	Caucasian	Cerebrovascular stroke	None
WK4	52 years	Female	Caucasian	Stroke	None

Tables S2. Cell viability (means ± SEM) of 2D-cultured PTECs in the presence of C12-, C14-, and C16-BAC parent compared to the ω-OH BAC metabolite over 48 hours (for Figure 2).

Concentration (μM)	C_{12} BAC	
	Parent	ω-OH
0	100±0.00	100±0.00
0.05	120.71±19.80	100.35±8.81
0.1	124.65±24.62	96.59±17.08
0.5	107.56±16.86	83.72±3.53
1	105.47±5.39	91.90±11.38
5	39.04±1.91	97.62±8.62
10	15.15±3.51	87.21±7.25
20	6.60±0.94	85.72±2.55
40	6.46±0.73	71.69±6.27

Concentration (μM)	C_{14} BAC	
	Parent	ω-OH
0	100±0.00	100±0.00
0.05	102.71±11.42	127.77±22.88
0.1	107.31±10.44	119.59±14.54
0.5	104.25±16.88	128.76±30.39
1	92.07±6.65	96.07±2.07
5	7.70±1.89	126.07±22.36
10	7.42±0.66	119.81±14.22
20	6.79±1.58	54.95±7.94
40	7.14±1.50	37.05±3.86

Concentration (μM)	C_{16} BAC	
	Parent	ω-OH
0	100±0.00	100±0.00
0.05	93.34±8.80	96.22±10.06
0.1	98.72±5.78	95.52±16.14
0.5	95.81±4.40	101.40±13.97
1	78.63±9.06	90.41±7.86
5	5.92±0.53	78.17±12.86
10	5.58±0.40	26.58±3.34
20	6.29±0.41	3.76±0.88
40	10.78±0.29	4.72±1.27

Tables S3. Cell viability (means ± SEM) of 2D-cultured PTECs treated with different concentrations of BACs in the presence or absence of CYP4F inhibitor, HET0016, for 48 hours (for Figure 3a).

Concentration (μM)	C_{12} BAC	
	no HET	HET
0	100±0.00	100±0.00
0.01	101.41+27.05	94.04±4.32
0.04	98.64±37.81	98.11±20.66
0.1	101.90±29.70	107.81±15.95
0.4	70.40±15.66	83.68±24.95
1	91.23±33.84	72.40±6.76
4	65.54±24.35	41.14±15.33
10	9.72±4.22	8.08±1.63
40	8.85±1.97	8.63±1.39
100	9.65±2.21	9.79±1.61
Concentration (μM)	C_{14} BAC	
	no HET	HET
0	100±0.00	100±0.00
0.01	123.54±11.19	82.76±17.79
0.04	124.67±4.57	81.95±16.18
0.1	125.63±19.50	80.56±30.52
0.4	112.25±14.30	86.67±22.61
1	89.65±5.72	68.85±8.14
4	8.40±2.65	25.11±32.93
10	8.75±2.51	6.73±0.72
40	11.07±3.77	8.56±1.15
100	19.97±3.85	9.93±1.86

Concentration (μM)	C_{16} BAC	
	no HET	HET
0	100±0.00	100±0.00
0.01	115.19±11.30	96.06±12.25
0.04	104.04±7.48	88.15±13.79
0.1	95.40±6.75	92.73±22.61
0.4	89.56±2.57	86.44±14.28
1	79.86±16.13	79.13±9.79
4	7.90±0.46	6.89±0.24
10	8.48±0.51	8.47±0.90
40	14.70±0.55	15.25±1.21
100	15.21±2.22	13.90±2.28

Table S4. Solvent gradients of LC-MS targeted analysis of BACs and metabolites.

	Gradient 1		Gradient 2		Gradient 3	
Time (min.)	A (%)	B (%)	A (%)	B (%)	A (%)	B (%)
0	80	20	75	25	70	30
10	20	80	15	85	20	80
11-12	0	100	0	100	0	100
12.5-14	80	20	75	25	70	30

Table S5. Precursor ion transitions and analyte retention times of parent BACs and metabolites[1].

Analyte	m/z	Retention Time (min.)	Gradient
diOH C12-BAC	336.287+244.225+91.054	2.91	1
(ω-1)-OH C12-BAC	320.295+228.233+91.055	4.24	1
COOH C12-BAC	334.271+242.212+91.054	4.28	1
ω-OH C12-BAC	320.295+228.233+91.054	4.41	1
ketone C12-BAC	318.277+226.217+91.054	4.55	1
C8-BAC	248.236+156.174+91.054	5.01	1
d_7-C12 BAC	311.342+212.238+98.098	8.01	1
C12 BAC	336.287+244.225+91.054	8.03	1
diOH C14-BAC	364.322+272.258+91.054	5.47	2
C8-BAC	248.236+156.174+91.054	6.56	2
COOH C14-BAC	362.306+270.242+91.054	7.11	2
(ω-1)-OH C14-BAC	348.326+256.264+91.054	7.16	2
ω-OH C14-BAC	348.326+256.264+91.054	7.37	2
ketone C14-BAC	346.311+254.247+91.054	7.58	2
d_7-C14 BAC	339.377+240.268+98.098	10.09	2
C14 BAC	346.311+254.247+91.054	10.11	2
C8-BAC	248.236+156.174+91.054	3.7	3
diOH C16-BAC	392.350+300.292+91.054	3.86	3
COOH C16-BAC	390.338+298.277+91.054	5.50	3
(ω-1)-OH C16-BAC	376.355+284.293+91.054	5.67	3
ω-OH C16-BAC	376.355+284.293+91.054	5.93	3
ketone C16-BAC	374.344+282.28+91.054	6.10	3
d_7-C16 BAC	367.405+268.300+98.098	10.51	3
C16 BAC	360.365+268.300+91.054	10.53	3

[1] Limit of detection (LOD) was calculated as 0.66 nM.

Table S6. Parent BAC and metabolite for C_{12}, C_{14} and C_{16} BAC in Figure 3b and 3c (values are in pmol per 5000 cells; SD = standard deviation).

0.4 μM treatment:

Compound	No HET (mean ± SD)	HET (mean ± SD)
C_{12}-BAC	5.51±0.07	17.53±1.90
ω-OH-C_{12}-BAC	0.94±0.35	0.08±0.02
(ω-1)-OH-C_{12}-BAC	0.15±0.01	0.02±0.00
COOH-C_{12}-BAC	0.34±0.01	0.04±0.01
(ω-1)-ketone-C_{12}-BAC	0.17±0.02	0.03±0.01
C_{14}-BAC	8.39±3.75	15.31±1.28
ω-OH-C_{14}-BAC	0.59±0.41	0.04±0.01
(ω-1)-OH-C_{14}-BAC	0.11±0.05	0.03±0.01
COOH-C_{14}-BAC	0.09±0.06	0.02±0.01
(ω-1)-ketone-C_{14}-BAC	0.06±0.02	0.01±0.01
C_{16}-BAC	7.01±2.25	7.32±0.73
ω-OH-C_{16}-BAC	7.20±0.35	0.04±0.02
(ω-1)-OH-C_{16}-BAC	0.50±0.06	0.03±0.03
COOH-C_{16}-BAC	1.16±0.18	0.01±0.01
(ω-1)-ketone-C_{16}-BAC	0.21±0.01	0.02±0.02
C_{16}-BAC-(ω,ω-1)-diol	0.05±0.01	0.00±0.00

1 μM treatment:

Compound	No HET (mean±Sdev)	HET (mean±Sdev)
C_{12}-BAC	20.88±5.99	47.66±10.72
ω-OH-C_{12}-BAC	1.30±0.49	0.14±0.01
(ω-1)-OH-C_{12}-BAC	0.25±0.04	0.06±0.02
COOH-C_{12}-BAC	0.59±0.05	0.10±0.04
(ω-1)-ketone-C_{12}-BAC	0.29±0.03	0.04±0.04
C_{14}-BAC	25.67±1.25	41.80±3.61
ω-OH-C_{14}-BAC	0.77±0.33	0.08±0.01
(ω-1)-OH-C_{14}-BAC	0.15±0.03	0.04±0.02
COOH-C_{14}-BAC	0.18±0.06	0.03±0.01
(ω-1)-ketone-C_{14}-BAC	0.08±0.01	0.02±0.02
C_{16}-BAC	15.47±3.43	19.41±0.74
ω-OH-C_{16}-BAC	12.35±0.49	0.08±0.04
(ω-1)-OH-C_{16}-BAC	0.79±0.05	0.04±0.01
COOH-C_{16}-BAC	1.65±0.09	0.01±0.00
(ω-1)-ketone-C_{16}-BAC	0.39±0.01	0.01±0.00
C_{16}-BAC-(ω,ω-1)-diol	0.10±0.02	0.00±0.00

Table S7. Sterol retention times, MS/MS transitions, and concentrations.

IS = Internal Standard
Std Conc.= Standard Concentration
Q = Quadrupole

**Analyte**	**Retention Time (min)**	**Q1**	**Q3**	**Std Conc. Std+IS Mix**	**IS Used**	**IS Conc. Std+IS Mix**
7-Dehydrodesmosterol	5.18	365.3	365.3	0.2 μg/mL	$^{13}C_3$-desmosterol	0.2 μg/mL
Zymosterol	7.20	367.3	367.3	0.2 μg/mL	$^{13}C_3$-lanosterol	0.2 μg/mL
Desmosterol	7.60	367.3	367.3	0.2 μg/mL	$^{13}C_3$-desmosterol	0.2 μg/mL
8-Dehydrocholesterol	8.17	367.3	367.3	0.2 μg/mL	d_7-7-dehydrocholesterol	1.0 μg/mL
7-Dehydrocholesterol	8.37	367.3	367.3	0.2 μg/mL	d_7-7-dehydrocholesterol	1.0 μg/mL
Cholesterol	10.60	369.3	369.3	0.2 μg/mL	d_7-cholesterol	1.0 μg/mL
Lanosterol	12.30	409.3	409.3	0.2 μg/mL	$^{13}C_3$-lanosterol	0.2 μg/mL

Table S8. Retention times and MS/MS transitions for sterol internal standards.

Internal Standard	Retention Time (min)	Q1	Q3
$^{13}C_3$-desmosterol	7.60	370.3	370.3
d_7-7-dehydrocholesterol	8.40	374.3	374.3
d_7-cholesterol	10.60	376.3	376.3
$^{13}C_3$-lanosterol	12.30	412.3	412.3

Table S9. Source and MS conditions for sterolomics.

APCI Conditions

Capillary Voltage	3 kV
Cone Voltage	20 V
Desolvation Temperature	250°C
Source Temperature	150°C
Desolvation Gas Flow	600 L/Hr
Cone Gas Flow	150 L/hr
Nebulizer Gas Flow	4.0 Bar
Collision Gas Flow	0.15 mL/min

MS Conditions

Collision Energy	5 V
Cone Voltage	10 V
Entrance Potential	30 V
Exit Potential	30 V

Table S10. Top 10 differentially expressed pathways in C_{12}- or C_{14}-BAC-treated MPS.

C_{12}-BAC

**Pathways**	**-log(p-value)**
Complement and coagulation cascades	3.961
Fat digestion and absorption	2.523
Focal adhesion	1.921
Glutathione metabolism	1.796
Relaxin signaling pathway	1.796
Arrhythmogenic right ventricular cardiomyopathy	1.586
Tight junction	1.509
ECM-receptor interaction	1.495
Dilated cardiomyopathy	1.481
Intestinal immune network for IgA production	1.432

C_{14}-BAC

**Pathways**	**-log(p-value)**
Viral protein interaction with cytokine and cytokine receptor	4.750
Cytokine-cytokine receptor interaction	4.570
Lysosome	4.077
Complement and coagulation cascades	3.758
Chemokine signaling pathway	3.335
Focal adhesion	3.187
Metabolic pathways	3.048
ECM-receptor interaction	2.699
Protein digestion and absorption	2.523
Cell adhesion molecules	2.523

Table S11. Top 10 differentially expressed genes in C12 and C14 BAC-treated MPS.

C12 BAC

Gene	Description	LogFC	P-value
KLHL4	Kelch like family member 4	-3.498	0.098
SURF1	SURF1 cytochrome c oxidase assembly factor	-1.829	0.019
LTF	Lactotransferrin	-1.649	1.763e-4
SERPINF2	Serpin family F member 2	-1.327	0.057
NAT8	N-acetyltransferase 8 (putative)	-1.223	0.082
RXFP1	Relaxin family peptide receptor 1	1.183	0.026
SCEL	Sciellin	0.774	0.067
COL1A1	Collagen type I alpha 1 chain	0.692	0.080
HPGD	15-Hydroxyprostaglandin dehydrogenase	0.619	0.067
FNIP2	Folliculin interacting protein 2	0.600	0.020

C14 BAC

Gene	Description	LogFC	P-value
SLC22A6	Solute carrier family 22 member 6<br>Rh family B glycoprotein	-3.535	0.007
RHBG	Rh family B glycoprotein	-3.442	0.015
APOM	Apolipoprotein M	-3.300	0.066
SLC13A1	Solute carrier family 13 member 1	-2.877	0.011
COL3A1	Collagen type III alpha 1 chain	-2.763	0.062
PPBP	Pro-platelet basic protein	3.828	0.025
CEACAM5	CEA cell adhesion molecule 5	3.648	0.003
IDO1	Indoleamine 2,3-dioxygenase 1	3.238	0.021
S100P	S100 calcium binding protein P	2.674	0.015
CXCL5	C-X-C motif chemokine ligand 5	2.566	0.021

Table S12. Cell cycle distribution from flow cytometry analysis of BAC treatments.

Sample	% G1	% S	% G2	RMSD
Negative control_1	44.3	41.8	4.47	1.67
Negative control_2	44.7	38.1	4.38	3.18
Negative control_3	52.0	20.2	3.57	4.91
C12 BAC_1	3.81	86.9	3.93	0.24
C12 BAC_2	15.1	73.2	4.25	0.85
C12 BAC_3	16.1	72.1	3.85	0.98
C14 BAC_1	18.7	70.9	3.47	0.69
C14 BAC_2	12.8	78.5	3.12	0.71
C14 BAC_3	13.5	76.7	3.65	1.09

Table S13. Cell cycle distribution from flow cytometry of negative and positive controls.

Sample	% G1	% S	% G2	RMSD
Negative control_1	43.4	40.6	7.51	2.60
Negative control_2	51.9	32.3	7.91	2.60
Negative control_3	48.6	35.9	7.44	2.83
Nocodazole_1	14.7	44.8	25.0	2.11
Nocodazole_2	9.87	53.7	20.2	2.12
Nocodazole_3	9.67	55.1	18.5	1.84
Thymidine_1	7.78	79.7	3.73	1.38
Thymidine_2	7.06	80.0	3.81	1.33
Thymidine_3	8.91	77.1	4.79	1.30

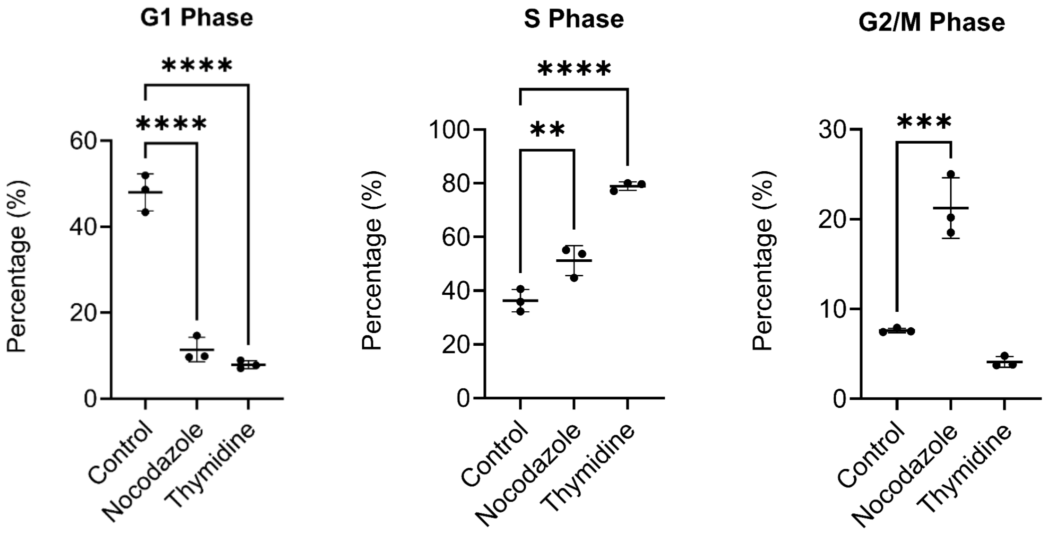

Figure S1. Cell cycle distribution from flow cytometry analysis of positive controls. N = 3 per group. *, $P < 0.05$; **, $P < 0.005$; ***, $P < 0.0005$; ****, $P < 0.00005$.

Tables S14. Quantitative data for Figure 7A in Weber et al., *Kidney Int*. 2016, 90, 627: 1,25-$(OH)_2$-Vitamin D_3, 4β,25−$(OH)_2$-Vitamin D3, 24,25-$(OH)_2$-Vitamin D_3 means, SEM, SD, and coefficient of variations (CV).

1,25-$(OH)_2$-Vitamin D3

	1,25-$(OH)_2$-Vitamin D3			
Day	mean	SEM	SD	CV (%)
0.5	0.00159	0.00029	0.00050	31.3
1.5	0.00341	0.00056	0.00097	28.6
2.5	0.00340	0.00055	0.00096	28.3

4β,25−$(OH)_2$-Vitamin D3

	4β,25−$(OH)_2$-Vitamin D3			
Day	mean	SEM	SD	CV (%)
0.5	0.00210	0.00041	0.00071	33.6
1.5	0.00318	0.00041	0.00071	22.3
2.5	0.00359	0.00052	0.00090	25.2

24,25-$(OH)_2$-Vitamin D3

	24,25-$(OH)_2$-Vitamin D3			
Day	mean	SEM	SD	CV (%)
0.5	0.0170	0.0014	0.0023	13.7
1.5	0.0220	0.0012	0.0021	9.5
2.5	0.0231	0.0033	0.0057	24.7

Tables S15. Quantitative data for Figure 7B in Weber et al., *Kidney Int*. 2016, 90, 627: Calcidiol and calcidiol+calcitriol SEM, SD, and coefficient of variations (CV).

	Calcidiol Only			Calcidiol + Calcitriol		
Day	mean	SD	CV (%)	mean	SD	CV (%)
0.5	0.0302	0.0057	18.9	0.0899	0.0294	32.7
1.5	0.0306	0.0071	23.4	0.1024	0.0163	15.9
2.5	0.0288	0.0058	20.0	0.1133	0.0187	16.5
